# Depletion of *SNORA33* Abolishes ψ of 28S-U4966 and Affects the Ribosome Translational Apparatus

**DOI:** 10.3390/ijms241612578

**Published:** 2023-08-08

**Authors:** Alzbeta Chabronova, Guus van den Akker, Bas A. C. Housmans, Marjolein M. J. Caron, Andy Cremers, Don A. M. Surtel, Mandy J. Peffers, Lodewijk W. van Rhijn, Virginie Marchand, Yuri Motorin, Tim J. M. Welting

**Affiliations:** 1 Laboratory for Experimental Orthopedics, Department of Orthopedic Surgery, Maastricht University, 6229 HX Maastricht, The Netherlands; a.chabronova@maastrichtuniversity.nl (A.C.); b.housmans@maastrichtuniversity.nl (B.A.C.H.);; 2 Institute of Life Course and Medical Sciences, University of Liverpool, Liverpool L8 7TX, UK; 3 UAR2008 IBSLor CNRS-INSERM-Université de Lorraine, F54000 Nancy, France; 4 UMR7365 IMOPA, CNRS-Université de Lorraine, F54000 Nancy, France; 5 Laboratory for Experimental Orthopedics, Department of Orthopedic Surgery, Maastricht University Medical Center+ (MUMC+), 6229 HX Maastricht, The Netherlands

**Keywords:** ribosomal RNA, 28S, epitranscriptome, ribosome, chondrocytes, osteoarthritis

## Abstract

Eukaryotic ribosomes are complex molecular nanomachines translating genetic information from mRNAs into proteins. There is natural heterogeneity in ribosome composition. The pseudouridylation (ψ) of ribosomal RNAs (rRNAs) is one of the key sources of ribosome heterogeneity. Nevertheless, the functional consequences of ψ-based ribosome heterogeneity and its relevance for human disease are yet to be understood. Using HydraPsiSeq and a chronic disease model of non-osteoarthritic primary human articular chondrocytes exposed to osteoarthritic synovial fluid, we demonstrated that the disease microenvironment is capable of instigating site-specific changes in rRNA ψ profiles. To investigate one of the identified differential rRNA ψ sites (28S-ψ4966), we generated *SNORA22* and *SNORA33* KO SW1353 cell pools using LentiCRISPRv2/Cas9 and evaluated the ribosome translational capacity by ^35^S-Met/Cys incorporation, assessed the mode of translation initiation and ribosomal fidelity using dual luciferase reporters, and assessed cellular and ribosomal proteomes by LC-MS/MS. We uncovered that the depletion of *SNORA33*, but not *SNORA22*, reduced 28S-ψ4966 levels. The resulting loss of 28S-ψ4966 affected ribosomal protein composition and function and led to specific changes in the cellular proteome. Overall, our pioneering findings demonstrate that cells dynamically respond to disease-relevant changes in their environment by altering their rRNA pseudouridylation profiles, with consequences for ribosome function and the cellular proteome relevant to human disease.

## 1. Introduction

The eukaryotic ribosome represents the core cellular translation machinery. This complex cellular nanomachine consists of ribosomal proteins and ribosomal RNAs (rRNAs) [1]. Ribosomal RNAs are heavily post-transcriptionally modified [2]. Up to 228 modified nucleotides (post-transcriptional modifications, PTMs) have been reported in human 18S, 5.8S, and 28S rRNAs and this number keeps growing [2,3,4]. Pseudouridylation, with up to 104 already reported rRNA sites, is one of the most abundant rRNA modifications (exceeded only by 2′-*O*-methylation with ~110 sites) [3,4]. The pseudouridylation (ψ) of rRNA nucleotides is carried out by snoRNA–protein complexes (snoRNPs) composed of H/ACA snoRNAs and four core protein co-factors, NHP2, NOP10, GAR1, and Dyskerin (DKC1) [5,6,7]. The box H/ACA snoRNAs (SNORAs) site-directionally guide the pseudouridylation of specific nucleotides by base-pairing with the target rRNA sequence, allowing the pseudouridine synthase DKC1 to catalyze the isomerization of the target uridine to pseudouridine [5,6,7]. Many rRNA ψ sites are conserved among prokaryotic and eukaryotic species and are clustered in functionally important ribosomal regions that interact with tRNAs and mRNAs, including the peptidyl transferase center (PTC), the polypeptide exit tunnel, or the intersubunit bridge [2,8]. During evolution and with growing organismal complexity, the rRNA ψ content increased, presumably to reinforce the structural and functional stability of ribosomes [2,8]. Higher eukaryotes, especially mammals, have several additional rRNA ψ sites, usually located in the exterior regions of the ribosome [2,3]. 

PTMs of rRNAs are a major source of ribosome heterogeneity, with implications for ribosome functional specialization [9,10,11,12,13,14,15,16]. The first link between the ψ of rRNAs and human disease was made when mutations in the *DKC1* gene were found to be implicated in the pathogenesis of X-linked dyskeratosis congenita (X-DC) [17,18]. X-DC is a rare, inherited, multisystemic syndrome characterized by bone marrow failure, reticular skin pigmentation, and oral leukoplakia, and it is associated with a high risk of cancer [19]. Studies using cells from X-DC patients, hypomorphic *Dkc1* mutant mice, and a yeast strain with mutated *Cbf5p* (the yeast homologue of *DKC1*) reported deregulated translation, especially in connection to internal ribosome entry site (IRES)-mediated translation initiation [12,13]. A loss of Ψ in helix 69 of 28S rRNA, a highly conserved hotspot for rRNA PTMs that interacts with A- and P-site tRNAs and forms the intersubunit ribosome bridge, resulted in impaired growth, reduced amino acid incorporation, and defective ribosome subunits association, as well as faulty reading frame maintenance and stop codon recognition in yeast [20,21,22,23]. *DKC1* is highly expressed in a variety of cancer tissues and high *DKC1* expression correlates with poor prognosis [24]. Furthermore, the expression of several H/ACA snoRNAs is altered in cancer [25] and oncogene RAS was shown to be involved in the regulation of their expression (e.g., *SNORA23*, *SNORA24*, *SNORA26*, *SNORA48*, and *SNORA6*) [26]. A high-throughput, deep-sequencing-based method (HydraPsiSeq) for systematic rRNA ψ mapping and quantification was developed recently and allowed the first comprehensive analysis of ψ-based ribosome heterogeneity in human cells and the identification of a stable and variable group of ψ modifications [3]. Nevertheless, the roles of rRNA ψ-based ribosome heterogeneity in human disease are largely unknown. 

Osteoarthritis (OA) is the most common degenerative joint disease and a major cause of pain and disability in adults [27]. Osteoarthritis represents an active disease process that affects the entire joint and all its tissues (e.g., articular cartilage, synovium, or subchondral bone) [28,29]. Disturbances in the homeostasis of joint tissues create a pathological, degenerative joint microenvironment. Synovial fluid located in the joint cavity is a plasma ultrafiltrate enriched in molecules (e.g., proteins, lipids, or metabolites) secreted by joint tissues, and as such it reflects systemic and local pathological changes that occur during osteoarthritis [30,31,32]. Chondrocytes, cells residing in the cartilage extracellular matrix, rely on synovial fluid for their nourishment and survival [33]. The major hallmark of osteoarthritis is the degeneration of the articular cartilage, which is a result of the “activation” of quiescent chondrocytes residing in the cartilage extracellular matrix [28]. Changes in chondrocyte activity and cellular phenotype are fueled by alterations in their protein expression programs [34]. Aberrations of ribosome biogenesis and activity have been reported in osteoarthritic chondrocytes [35]. However, very little is known about the role of ribosome heterogeneity and specialization in the pathobiology of this chronic disease. Our group recently published data demonstrating that osteoarthritic synovial fluid instigates site-specific changes in the 2′-*O*-me rRNA profiles of human chondrocytes and that these changes have consequences for ribosome function and translation [36]. In this work, we used primary human articular chondrocytes exposed to the osteoarthritic synovial fluid to investigate the effect of a chronic disease microenvironment on rRNA ψ profiles.

## 2. Results

### 2.1. A Chronic Disease Microenvironment Provokes Site-Specific Changes in rRNA ψ Profiles

**Figure 1:**
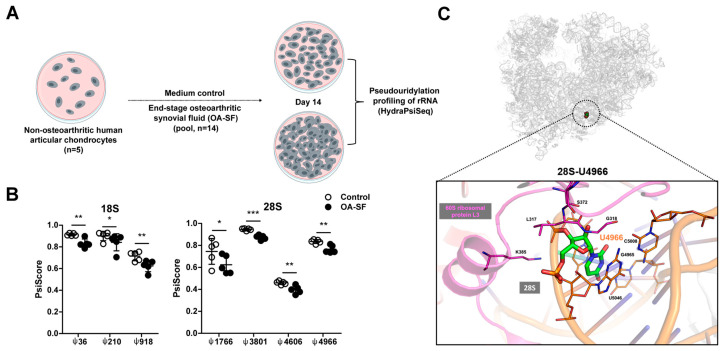
The chronic disease microenvironment induces site-specific changes in the pseudouridylation levels of ribosomal RNAs in primary human chondrocytes. (**A**) A schematic of the experimental design. Non-OA human articular chondrocytes of 5 individual donors were exposed to a chronic disease microenvironment represented by the synovial fluid of end-stage OA patients (OA-SF, pool of 14 donors, 20% (*v*/*v*)) for 14 days. The culture medium was refreshed every other day. After 14 days of culture, total RNA was isolated and used for the pseudouridylation profiling of rRNAs by HydraPsiSeq. (**B**) Differentially pseudouridylated (ψ) rRNA nucleotides (n = 5). Statistical significance was assessed by paired *t*-test with the assumption of a normal distribution of the data. Full ψ rRNA profiles are shown in Figure S1 and PsiScore values are listed in Table S1. (**C**) Location of 28S-U4966 within domain VI of the large ribosomal subunit of the human ribosome. * *p* < 0.05, ** *p* < 0.01, *** *p* < 0.001.

To explore the effect of a chronic disease microenvironment on rRNA ψ profiles, we employed an in vitro osteoarthritis disease model in which we exposed human primary chondrocytes to osteoarthritic synovial fluid (OA-SF) for 14 days and performed rRNA ψ HydraPsiSeq profiling (Figure 1A) [3]. The 14-day-long exposure of chondrocytes to OA_SF was chosen based on our previous data. Our time course experiment showed that the treatment with OA-SF triggered OA-related changes in chondrocytes’ gene expression and signaling from day 3 onwards, and these changes appeared to be rather stable until day 13 (the latest time point that we measured) [37]. In this study, we chose a time point of 14 days due to expected slow turnover of ribosomes when compared to gene expression changes. Ribosomal RNA ψ is considered irreversible, and the average half-life of rRNA in the cytoplasm of mouse articular cartilage is 5–7 days [38]. There are no conclusive data on the rRNA half-life in primary human chondrocytes; however, it could be expected that 14 days of OA-SF treatment would allow for the complete turnover of rRNAs (and ribosomes) and provide sufficient time for changes in rRNA ψ profiles to take place. We detected 102 pseudouridylated rRNA nucleotides (two sites not detected). Of these, we observed 41 in 18S, 2 in 5.8S, and 59 in 28S (Figure S1 and Table S1). We identified seven differentially pseudouridylated sites in the OA-SF-treated cells, three sites in 18S (18S-ψ36, 18S-ψ210, 18S-ψ918), and four sites in 28S (28S-ψ1766, 28S-ψ3801, 28S-ψ4606, and 28S-ψ4966) (Figure 1B). In particular, 18S-ψ36 and 18S-ψ210 are both located in domain 5’ of the small human ribosomal subunit, specifically within helix 4 (18S-ψ36) and helix 9es3a (18S-ψ210), while 18S-ψ918 is located in helix 21es6d/22 of domain C. The remaining four differentially modified sites are located within the large human ribosomal subunit, domain II H38a (28S-ψ1766), domain 0 H64/70 (28S-ψ3801), and domains VI H69 (28S-ψ4606) and H101 (28S-ψ4966). All seven differentially ψ rRNA sites exhibited decreased modification levels in response to OA-SF. These data are the first evidence that a chronic disease microenvironment can induce changes in rRNA ψ profiles. There was no apparent clustering of these differential osteoarthritis-sensitive sites in terms of ribosomal helices or domains. Interestingly, decreased modification levels of 28S-ψ4966 were previously also reported in the cells of patients with familial X-DC [2]. Nevertheless, the relevance of 28S-U4966 and its conversion to ψ for ribosome function has not been investigated. Therefore, we undertook functional validation for this modification in our further work. 

### 2.2. Depletion of SNORA33 but Not SNORA22 Reduces 28S-ψ4966 Levels

**Figure 2:**
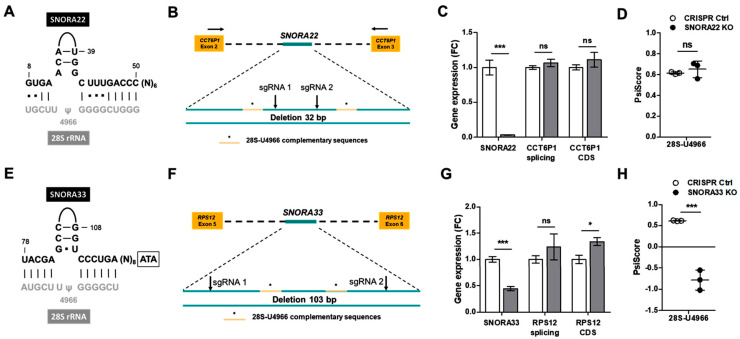
CRISPR/Cas9-mediated *SNORA33* depletion leads to the loss of pseudouridylation at 28S-U4966. (**A**,**E**) Two box H/ACA snoRNAs, SNORA22 and SNORA33, are predicted to guide the ψ of 28S-U4966. (**B**,**F**) A scheme of the double sgRNA CRISPR/Cas9-mediated approach to generate *SNORA22* and *SNORA33* SW1353 KO cell pools. Top: Exons of host genes *CCT6P1* and *RPS12* flanking the intron-encoded *SNORA22* and *SNORA33*, respectively. Bottom: Scheme of *SNORA22* and *SNORA33* sequences with indicated sgRNA1 and sgRNA2 cleavage sites and rRNA complementary sequences. The cell pool of GFP targeting CRISPR/Cas9-treated cells was used as a control (CRISPR Ctrl). (**C**) Expression levels of *SNORA22*, expression and splicing (exons 2 and 3) of its host gene *CCT6P1* measured by RT-qPCR (n = 3). (**G**) Expression levels of *SNORA33*, expression and splicing (exons 5 and 6) of its host gene *RPS12* measured by RT-qPCR (n = 3). Expression levels were normalized to the reference gene (PPIA) expression and plotted as fold change to CRISPR Ctrl. Data were analyzed by unpaired *t*-test with the assumption of a normal distribution. (**D**,**H**) Pseudouridylation levels of 28S-U4966 (n = 3) measured by HydraPsiSeq and analyzed by unpaired *t*-test with the assumption of a normal distribution of data. Negative PsiScore for 28S-ψ4966 in the case of *SNORA33* KO cell pool indicates even more intensive hydrazine cleavage at this position compared to other neighboring U residues. Ns – not significant, * *p* < 0.05, *** *p* < 0.001.

**Figure 3:**
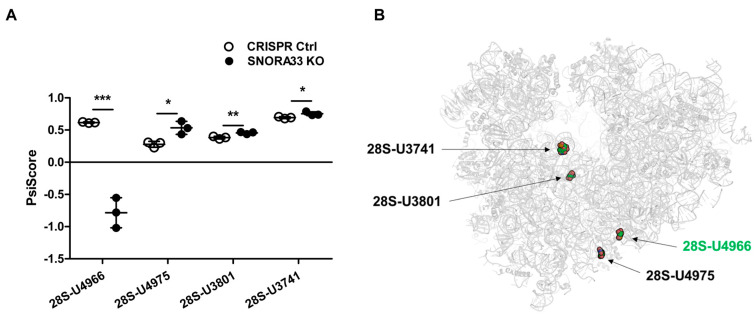
The effect of *SNORA33* depletion on rRNA ψ profile. (**A**) Differentially ψ rRNA nucleotides in *SNORA33*-depleted cells (n = 3). Statistical significance was assessed by unpaired *t*-test with the assumption of a normal distribution of data. Full ψ rRNA profiles are shown in Figure S4 and PsiScore values are listed in Table S2. (**B**) Negative PsiScore for 28S-ψ4966 in the case of *SNORA33* KO cell pool indicates even more intensive hydrazine cleavage at this position compared to other neighboring U residues. The locations of differentially ψ sites identified in *SNORA33*-depleted cell pools mapped within a human ribosome. 28S-U3801 and 28S-U3741 are located in domain IV, and 28S-U4975 and 28S-U4966 are situated within domain VI of the large ribosomal subunit. * *p* < 0.05, ** *p* < 0.01, *** *p* < 0.001.

To functionally investigate 28S-ψ4966 and its role(s) in ribosome function and osteoarthritis pathobiology, we depleted chondrocytic SW1353 cells of snoRNAs guiding this specific pseudouridylation. Two intron-encoded H/ACA snoRNAs, SNORA22 and SNORA33, were previously predicted to guide the ψ of 28S-U4966 (Figure 2A,E) [39]. We used CRISPR/Cas9 gene editing and a double sgRNA targeting approach (Figure 2B,F) to generate *SNORA22* and *SNORA33* knockout (KO) cell pools along with a GFP-targeting CRISPR control cell pool. The efficiency of the gene editing was verified using the surveyor nuclease assay (Figure S2), and genomic DNA sequencing confirmed a 32 basepair (bp) deletion in the *SNORA22* gene and 103 bp deletion in the *SNORA33* gene (Figure S2C). Despite the complete loss of *SNORA22* expression, we did not measure any decrease in 28S-ψ4966 levels (Figure 2C,D). We also did not measure any effects of *SNORA22* deletion on its host gene (*CCT6P1*) expression or splicing. On the other hand, a 55% decrease in *SNORA33* expression resulted in the ablation of ψ at 28S-U4966 (Figure 2G,H). These results imply that the ψ of 28S-U4966 is in fact guided only by SNORA33. Looking at the gene expression and splicing of the *SNORA33* host gene *RPS12*, we noted a slight increase in RPS12 coding sequence (CDS) levels, but no effect on the splicing of exons 5 and 6 enclosing the *SNORA33*-hosting intron region (Figure 2G). In conclusion, our data demonstrate that the ψ of 28S-U4966 is guided by SNORA33. Besides the ablation of 28S-ψ4966 in *SNORA33*-depleted cells, we also measured a modest, but statistically significant, increase in ψ levels at three other sites, 28S-ψ4975, 28S-ψ3801, and 28S-ψ3741 (Figure 3A and Figure S4, Table S2). By mapping the sites with differential ψ status in *SNORA33*-depleted cells within the 3D human ribosome, we observed that these sites clustered in domains IV and VI of the large ribosomal subunit (Figure 3B). While 28S-U3801 and 28S-U3741 are located in domain IV, 28S-U4975 and 28S-U4966 are situated within domain VI. Interestingly, the ψ levels of 28S-U3801 were also regulated (decreased) in our original in vitro chronic disease model on which rRNA ψ HydraPsiSeq profiling was applied (Figure 1A). To corroborate the relevance of SNORA33, and by extension the 28S-U4966 pseudouridylation levels in osteoarthritis, we measured the *SNORA33* expression levels in freshly isolated healthy and osteoarthritic human primary chondrocytes. Results showed that *SNORA33* expression was indeed significantly decreased in osteoarthritic chondrocytes compared to healthy controls (Figure S3). 

### 2.3. SNORA33 Depletion Affects Ribosome Composition, Function, and the Cellular Proteome

**Figure 4:**
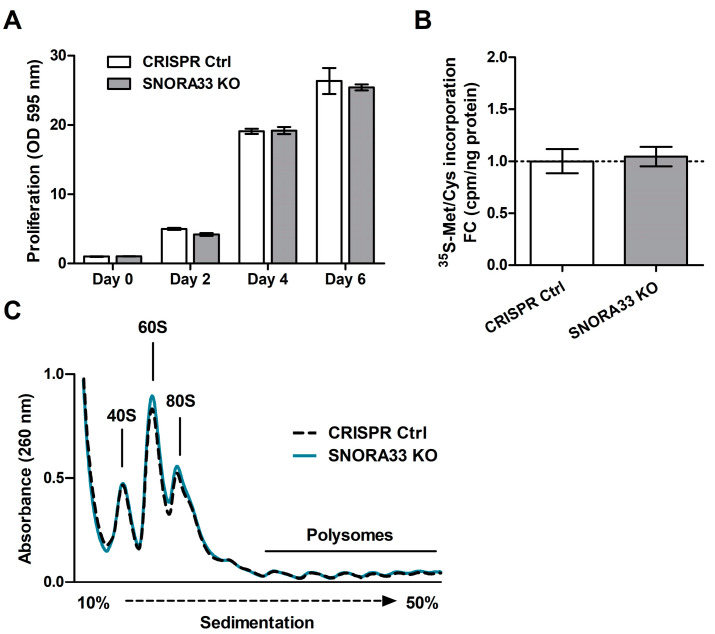
*SNORA33* depletion does not affect proliferation or translation capacity. (**A**) Growth rate of *SNORA33*-depleted cells measured by crystal violet staining. Data (n = 4) are presented as fold change to Day 0 and were analyzed by two-way ANOVA. (**B**) The total rate of protein synthesis of *SNORA33*-depleted cells (n = 3) measured by [^35^S]methionine/cysteine incorporation. Data are presented as fold changes to CRISPR control. Statistical significance was assessed by unpaired *t*-test with the assumption of a normal distribution of the data. (**C**) Polysome profiles of CRISPR control and *SNORA33*-depleted cells (n = 1).

**Figure 5:**
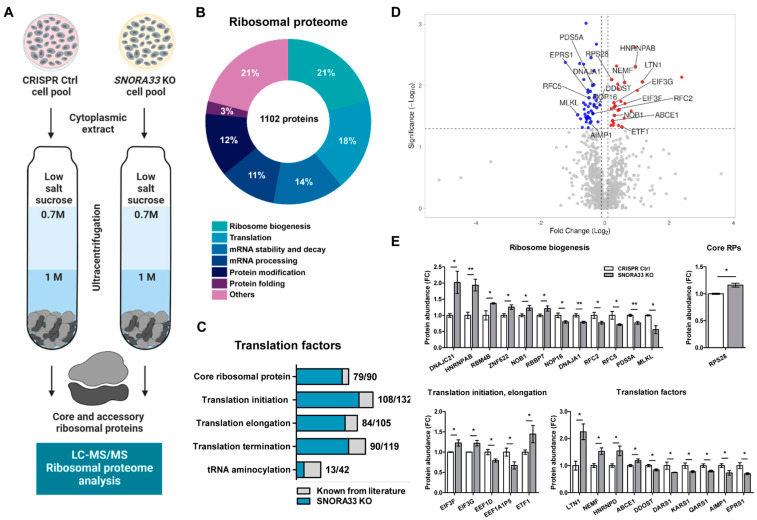
Depletion of *SNORA33* affects ribosomal protein composition. (**A**) The scheme of the experimental set-up. Cytoplasmic extracts of *SNORA33*-depleted and CRIRP Ctrl cells (n = 3) were depleted of mitochondria and nuclei and ultracentrifuged through the sucrose cushions. Ribosomal pellets were resuspended and analyzed by label-free LC-MS/MS. (**B**) A pie chart of all identified ribosomal proteins and their functional allocation. (**C**) Translation factors identified in the ribosomal proteome. (**D**) A volcano plot of all identified ribosomal proteins. Normal distribution of the data and equal variance of populations was assumed. Statistical significance was assessed by one-way ANOVA. The dotted lines represent cut-off values (FC ≥ 1.1; *p* < 0.05). Ribosomal proteins significantly less or more abundant in ribosomes of *SNORA33*-depleted cells are in red and blue, respectively. (**E**) Protein abundance of selection of differentially abundant core and accessory ribosomal proteins in *SNORA33* KO cell pool. Data are presented as fold changes to CRISPR control. * *p* < 0.05, ** *p* < 0.01.

After establishing the *SNORA33* KO cell pools, we proceeded with the functional validation of the relevance of 28S-ψ4966, by evaluating the ribosome composition and function in *SNORA33*-depleted cells. First, we probed whether the depletion of *SNORA33* affected overall cellular fitness, by assessing the proliferation rate, total protein translation, and polysomal distribution. We did not detect any differences in these characteristics in the *SNORA33*-depleted cells compared to CRISPR controls (Figure 4). Next, we examined the protein compositions of ribosomes devoid of 28S-ψ4966. We used low salt procedures [40] for the ultracentrifugation of cytoplasmic extracts through sucrose cushions to isolate ribosomes as well as ribosome-associated factors and accessory proteins (Figure 5A). Analysis of the ribosomal proteome identified 1102 proteins, including 79 core ribosomal proteins, many ribosome biogenesis factors, and a large number of translation initiation, elongation, and termination factors. We also identified proteins involved in mRNA processing, stability, and decay and factors assisting protein folding and modification (Figure 5B,C; analyzed using the Reactome [41]). Seventy-four proteins showed differential abundance in isolated ribosomes lacking 28S-ψ4966 (Figure 5D, Table S3). Among the differentially abundant proteins were several ribosome biogenesis factors (e.g., NOB1, DNAJA1, PDS5A), translation initiation factors EIF3F and EIF3G, elongation factor EEF1D, eukaryotic peptide chain release factor 1 (ETF1), and proteins involved in ribosome quality control, LTN1 and NEMF (Table S4, Figure 5E). A single core ribosomal protein, RPS28, showed increased association with ribosomes depleted of 28S-ψ4966 (Figure 5E). 

**Figure 6:**
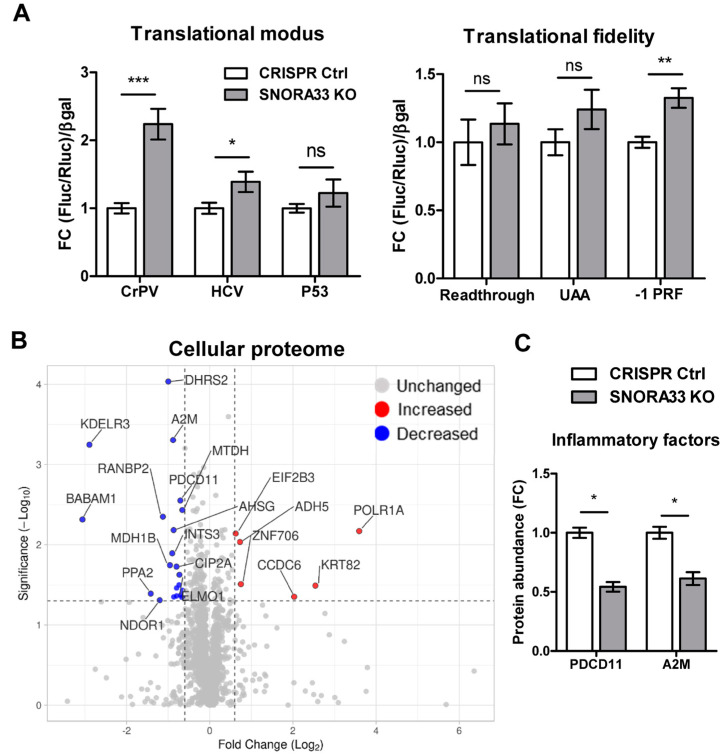
*SNORA33* depletion affects ribosome function and cellular proteome. (**A**) The effect of *SNORA33* depletion on translational modus and fidelity. Dual-luciferase reporters were used to measure the mode of translation initiation from IRES elements (CrPV, HCV, and P53) and translation fidelity (UAA—stop codon skipping, and −1 PRF—frameshift). Data (n = 9) are corrected for the β-galactosidase activity of the co-transfected lacZ gene and are plotted as fold changes to CRISPR Ctrl. The D’Agostino–Pearson test was used to assess normality and data were analyzed by a nonparametric Mann–Whitney U test. (**B**) LC-MS/MS analysis of the cellular proteome of *SNORA33*-depleted cells and CRISPR controls. A volcano plot of all proteins identified in the cellular proteome (n = 4). Normal distribution of the data and equal variance of populations were assumed. Statistical significance was assessed by one-way ANOVA. The dotted lines represent cut-off values (FC ≥ 1.5; *p* < 0.05). Cellular proteins significantly downregulated and upregulated in *SNORA33*-depleted cells are in red and blue, respectively. (**C**) Protein abundance of significantly DE inflammatory factors in *SNORA33* KO cell pool. Data are presented as fold changes to CRISPR control. Ns – not significant, * *p* < 0.05, ** *p* < 0.01, *** *p* < 0.001.

To examine whether ribosome heterogeneity based on the identified differential ribosomal protein composition and 28S ψ affects ribosome function, we probed the mode of translation initiation and translation fidelity in the *SNORA33* KO cell pool using dual-luciferase reporter assays. We demonstrated the increased capacity of 28S-ψ4966-depleted ribosomes to initiate translation from CrPV and HCV IRES elements (Figure 6A). Translation accuracy was also affected, as evidenced by a greater incidence of -1 frameshift activity. Subsequently, we analyzed the cellular proteome of *SNORA33*-depleted cells. We detected 2377 cellular proteins, of which 28 proteins were differentially expressed (DE) in *SNORA33*-depleted cells compared to CRISPR controls (6 upregulated and 22 downregulated, Figure 6B, Table S5). The most upregulated protein was a subunit of the RNA polymerase I complex, POLR1A, which catalyzes the synthesis of 18S, 5.8S, and 28S rRNAs (47S transcript) [42]. Several inflammation-related factors were among the DE proteins, including programmed cell death protein 11 (PDCD11, also called NFBP) and α-2-macroglobulin (A2M) (Figure 6C). Importantly, we did not detect differential expression of the SNORA33 host gene *RPS12.* Of the proteins that were found to be differentially associated with the ribosomes of *SNORA33*-depleted cells, none was found to be DE in the cellular proteome. This implies that the ribosomal proteome data are reflective of distinct associations of these proteins with ribosomes, rather than their expression. Combined, our results demonstrate that *SNORA33* depletion and the loss of 28S-ψ4966 affect ribosome composition and function, with consequences for the cellular proteome.

## 3. Discussion

The recent development of a high-throughput method for systematic ψ mapping, HydraPsiSeq, enabled a comprehensive analysis of ψ-based ribosome heterogeneity in human cells [3]. Nevertheless, the driving force behind the differential ψ of rRNA nucleotides and their functional consequences for human disease are largely unknown. We used an in vitro disease model to demonstrate that the rRNA pseudouridylation profiles of human primary cells are sensitive to disease-related changes in their microenvironment. We identified seven rRNA sites whose ψ-modification levels significantly decreased in response to a chronic osteoarthritis disease microenvironment. We selected one of them, 28S-ψ4966, for more detailed functional validation. Out of two snoRNAs (SNORA33 and SNORA22) that have been predicted to guide the ψ conversion of 28S-U4966, we determined that only SNORA33 is in fact responsible for this PTM in SW1353 cells. Depleting chondrocytic cells of *SNORA33* using a CRISPR/Cas approach, we generated a cell pool devoid of 28S-ψ4966 and proceeded with the ribosome- and translation-focused experiments. The ablation of *SNORA33* and thus 28S-ψ4966 affected the ribosomal protein composition, as evidenced by the differential association of core ribosomal protein RPS28 and many ribosome-associated factors related to translation initiation and elongation. Consequently, we detected alterations in ribosome translational modus and fidelity, as well as the differential expression of several inflammation-related proteins in the cellular proteome of the *SNORA33*-depleted SW1353 cells. 

Osteoarthritis is a degenerative chronic joint disease. Synovial fluid is a major determinant of the cartilage (and chondrocytes) microenvironment that well reflects the pathological processes occurring in the joint during osteoarthritis [30,31,32]. Recently, we performed a direct comparison of non-osteoarthritic and osteoarthritic synovial fluid and we identified critical differences in their compositions and effects on chondrocytes [43]. We showed that OA-SF is strongly enriched in inflammatory mediators, growth factors, and disease-associated molecular patterns (DAMPs) and provokes differential chondrocyte signaling, inflammatory responses, and chondrocyte phenotype alterations relevant to osteoarthritis. This demonstrates that chondrocytes exposed to osteoarthritic synovial fluid represent a relevant model to study how the local joint disease microenvironment affects the pathomolecular characteristics of chondrocytes [36,37,43]. In the current study, we did not use non-OA-SF as a control. Instead, we utilized a culture medium supplemented with 0.9% NaCl in the same *v/v* concentration as OA-SF. This approach was employed due to the limited availability of human non-OA-SF. Importantly, the NaCl control was previously shown to be suitable for this type of comparison [36,37]. 

Recently published data from our group demonstrated that osteoarthritic synovial fluid instigates site-specific changes (18S-Gm1447, 5.8S-Um14, 28S-Am3739, 28S-Am3846, and 28S-Um4590) in the 2′-*O*-me rRNA profiles of human primary chondrocytes, with consequences for their translation [36]. Here, we extended the dataset of osteoarthritis-sensitive rRNA PTMs for seven rRNA ψ sites (18S-ψ36, 18S-ψ210, 18S-ψ918, 28S-ψ1766, 28S-ψ3801, 28S-ψ4606, and 28S-ψ4966). These data are valuable not only for the osteoarthritis field but also for research on ribosome heterogeneity in general. Thus far, studies on rRNA PTM-based ribosome heterogeneity in humans have focused mainly on measuring differences between different cell lines, cell types, and healthy and diseased tissues [3,11], while only a handful of studies investigated the regulatory mechanisms behind the differential rRNA PTMs [16]. Data on rRNA ψ are particularly limited as a high-throughput method for systematic ψ quantification was only recently developed in 2020 [3]. Our data represent a pioneering study that analyzed the full ψ signature of human primary cells in response to a chronic disease microenvironment. The magnitude of changes in rRNA ψ profiles in response to chronic disease microenvironment was relatively small, ranging from 5.8 to 12.1%. However, these changes were significant and consistent across all five individual donors. Although further research is needed to evaluate the relevance of this scale of ribosome rRNA heterogeneity for human diseases, we believe that it might contribute to pathological changes in articular cartilage in osteoarthritis [35].

Lower modification levels of one of the osteoarthritis-sensitive rRNA ψ sites were previously measured in fibroblasts and B-lymphoblastoid cell lines derived from X-DC patients when compared to profiles of their unaffected relatives [2]. In fact, only two sites exhibited decreased ψ levels in the rRNAs of X-DC cells, 28S-ψ4331 and the here identified osteoarthritis-sensitive 28S-ψ4966. For this reason, we decided to follow up on this modification in further translation-focused experiments. 

Proteomic analysis of isolated ribosomes depleted of 28S-ψ4966 uncovered differential stoichiometry of several translation factors and a modest difference in the association of core ribosomal protein RPS28, which is located relatively far from 28S-ψ4966. RPS28 is part of the eukaryotic translation initiation complex and interacts with the 5’ UTRs of mRNAs [44]. Furthermore, it has been suggested that ribosomes containing RPS28 variants more efficiently translate mRNAs with certain 5’ or 3’ UTR features [45]. Interestingly, mutations in RPS28 have been implicated in Diamond–Blackfan anemia, a ribosomopathy characterized by red blood cell aplasia, skeletal anomalies, and short stature [46]. We speculate that the change in the association of RPS28 with the ribosomes of *SNORA33*-depleted cells might have contributed to the changes in ribosome function and cellular proteome that we observed. Nevertheless, a connection between *SNORA33* depletion and the stoichiometry of RPS28 remains elusive. Besides RPS28, we also found a differential association of multiple translation factors regulating various steps of translation, including translation initiation and elongation but also termination, as well as ribosome stalling and ribosome recycling. These unique data accentuate the mutually dependent relationships between rRNA PTMs and protein-based features of ribosome heterogeneity. 

IRES-mediated translation represents an alternative route of translation initiation that bypasses the cap-dependent mRNA scanning and directly binds the 40S ribosomal subunit to the vicinity of the start codon to initiate the translation in a cap-independent manner [47]. Although first discovered in viruses, the IRES-mediated translation of cellular mRNAs is becoming increasingly recognized as a mechanism activated under cellular stress [48]. The regulation behind the IRES-mediated translation in cellular systems is mostly incompletely understood. However, rRNA ψ is one of the few factors previously shown to influence the IRES-dependent translation of cellular mRNAs [12,15]. Studies of *Dkc1* hypomorphic mice and the cells of X-DC patients showed that not only do they have distinct ψ profiles but they also exhibit altered translation of specific mRNAs harboring IRES elements, such as *Bcl-xL*, *Xiap*, *p27*, *p53*, or *Vegf* [2,13,49,50,51]. Generally, IRES elements require the help of certain canonical translation factors and/or specific cellular IRES-transacting factors (ITAFs) [52]. A heterogeneous ribonucleoprotein D (HNRNPD, also known as AU-rich element RNA-binding protein 1 (AUF1)) is an ITAF that was found to interact with the HCV IRES and thus promote translation from the HCV IRES element [52]. We found the increased association of HNRNPD with the ribosomes of *SNORA33*-depleted cells, which agrees with our IRES reporter data and increased translation from the HCV IRES. As the 28S-ψ4966 and ribosome IRES-binding site are located relatively far from each other (Figure S5), we suspect that the observed changes in translation initiation are mediated by ITAFs differentially associating with ribosomes. Besides the translational modus, we also evaluated the frequency of the errors occurring during the translation. Knockout of RAS-regulated *SNORA24* and the decreased ψ of its rRNA targets 18S-U609 and 18S-U863 led to perturbations in aminoacyl-transfer RNA (aa-tRNA) selection, altered dynamics of the pre-translocation ribosome complex, and increased frequency of translational miscoding and stop codon readthrough [26]. Using a reporter construct based on the HIV-1 PRF sequence, we measured a higher frequency of −1 frameshifting in the *SNORA33*-depleted cells. Interestingly, ETF1 (also called eRF1), which was shown to decrease the incidence of the frameshift and thus inhibit HIV replication [53,54,55], was more abundantly associated with ribosomes lacking 28S-ψ4966. This may indicate the engagement of some compensatory mechanisms. Altogether, our data suggest that the modification level of 28S-ψ4966 affects ribosome protein stoichiometry, with consequences for translation regulation. In line with this, we also measured specific changes in the cellular proteome of the *SNORA33*-depleted cells, including decreased expression of several inflammation-regulating factors. One of them was PDCD11, also known as NFBP, which binds NF-κB subunits p50 and p65 and suppresses the expression of inflammatory cytokines [56,57]. A2M is an extracellular macromolecule that functions as a broad-spectrum protease inhibitor. A2M is expressed in articular cartilage and it is also present in synovial fluid [58]. It works as a negative regulator of cartilage catabolic enzymes and therefore inhibits cartilage degeneration [58]. Intraarticular injections of A2M were shown to attenuate the pathogenesis of post-traumatic osteoarthritis in the rat OA model [58,59]. Furthermore, a recent paper demonstrated that A2M binds and neutralizes IL-1β and thus inhibits the downstream NF-κB inflammatory signaling [60]. In this way, A2M inhibits the expression of cartilage-degenerating matrix metalloproteinases (MMPs) and TNFα, while it increases the expression of cartilage protective genes including collagen type II or aggrecan [60]. Inflammation plays a crucial role in osteoarthritis pathobiology [61]. Chondrocytes respond to pro-inflammatory factors in their microenvironment by downregulating their anabolic activities and, in contrast, upregulating the catabolic processes that eventually lead to cartilage degeneration. The downregulation of factors attenuating cellular inflammatory responses in *SNORA33*-depleted cells suggests that ribosome heterogeneity and the loss of 28S-ψ4966 promote chondrocyte inflammatory responses in osteoarthritis. In the future, more advanced translation measurements, such as Ribo-seq or SILAC mass-spec proteomics, would provide additional details about preferential translation regulation in chondrocytes in health and disease. 

A close inspection of the full ψ profile of *SNORA33*-depleted KO cell pools revealed that besides the ablation of 28S-ψ4966, this also led to a minor increase in ψ levels at three other sites on 28S (28S-U4975, 28S-U3801, 28S-U3741). All four sites were affected by the *SNORA33* depletion cluster within two regions of the large ribosomal subunit, domains IV (28S-U3801 and 28S-U3741) and VI (28S-U4975 and 28S-U4966). The concurrent regulation of ψ located close to each other could suggest steric effects. For example, the loss of 28S-ψ4966 might result in more space for the H/ACA snoRNP machinery to catalyze the isomerization of uridine at nearby 28S-U4975. Compensatory or competitive relationships cannot be excluded either. However, at this point, we know very little about the interrelationships and mutual regulation of individual rRNA PTMs to draw any definitive conclusion. 

The manipulation of snoRNA levels is a commonly used approach to control the levels of specific rRNA PTMs and to investigate the roles of rRNA-based ribosome heterogeneity in cellular phenotypes and ribosome functions. However, it has certain limitations, mostly due to the potential non-canonical functions of certain snoRNAs. For this reason, the conclusions of these studies need to be interpreted carefully, considering both the direct effects of the loss of PTMs guided by a given snoRNA and its possible indirect (non-canonical) functions. To the best of our knowledge, no non-canonical functions have been reported for SNORA33. Furthermore, we presented data connecting the 28S-ψ4966 modification levels with changes in ribosomal protein composition and thus linking it with the observed ribosome (dys)function and cellular disease phenotype.

Taken together, we present the first data showing that a chronic disease microenvironment instigates site-specific changes in the rRNA ψ profiles of human primary cells. The ablation of a single, disease-sensitive ψ site, 28S-ψ4966, affected ribosome composition and function, with consequences for the cellular proteome, relevant for human disease. Our data broaden the knowledge of ribosome heterogeneity, specialization, and their relevance for human pathologies.

## 4. Materials and Methods

### 4.1. Human Articular Chondrocytes Isolation and Culture

Non-OA primary human articular chondrocytes (HACs) were isolated from the cartilage of patients undergoing arthroscopy, anterior cruciate ligament repair, or osteochondritis dissecans surgery according to the previously described procedure [62]. Material collection and use were approved by the METC from the Maastricht University Medical Center (approval number 2017-0183) and informed consent was acquired from all subjects. Non-OA HACs (n = 5, individual donors) were plated at 30.000 cells/cm^2^ in DMEM/F-12 low glucose with GlutaMAX (Gibco Life Technologies, Waltham, MA, USA, 31331-093), 10% fetal calf serum (FCS; Sigma-Aldrich, Zwijndrecht, The Netherlands, F7524), 1% antibiotic–antimycotic (A-A, Gibco Life Technologies, 15240-062), and 1% nonessential amino acids (NEAA; Gibco Life Technologies, 11140-035). The day after seeding, cells were treated with 20% (*v*/*v*) OA-SF (equal volume ratios of OA-SF from 14 OA patients to minimize interpatient variability) or 20% (*v*/*v*) 0.9% NaCl. The treatment lasted for 14 days and the medium was refreshed every other day. 

### 4.2. Synovial Fluid Collection

Osteoarthritic synovial fluid was collected from patients undergoing total knee replacement surgery (n = 14, average age 67.1 ± 5.5 years) and stored at −80 °C until further use. Ethical approval was obtained from the Medical Ethics Committee (METC) at the Maastricht University Medical Center (approval number 2017-0183).

### 4.3. RNA Isolation and HydraPsiSeq

Total RNA was isolated by the RNeasy Plus Mini Kit (Qiagen, Manchester, UK, 74134) and its quality was determined with an RNA 6000 Nano Kit (Agilent, Abcoude, The Netherlands, 5067-1511) and 2100 Bioanalyzer (Agilent). All samples had an RNA integrity (RIN) number greater than 9. HydraPsiSeq of rRNAs was performed as previously described [3]. Total RNA was randomly fragmented at uridine residues by hydrazine (pseudouridines are resistant) and then treated with aniline for RNA scission at abasic sites. Fragments were converted into a library (NEBNext Small RNA Library kit), multiplexed, and sequenced by the Illumina HiSeq 1000 instrument (Illumina, Evry, France). The presence of pseudouridines was detected as the protection of modified U residues against hydrazine cleavage. The depth of the “gap” in the U-protection profile is proportional to the modification level and allows the calculation of a quantitative PsiScore. The value of PsiScore 1.0 represents a fully modified ψ position, while unmodified U shows a PsiScore close to 0 or even a negative value if more intensively cleaved compared to the neighboring U residues.

### 4.4. Visualization of the Human Ribosome and PTM Mapping

PTM sites were visualized using the PyMOL software 2.5 (Warren L. DeLano, sourced in Maastricht, The Netherlands) and the 3D structure of the human 80S ribosome from the Protein Data Bank (PDB ID: 6QZP) [63,64].

### 4.5. Lentiviral CRISPR/Cas9 Targeting snoRNAs

LentiCRISPR v2 (Addgene plasmid #52961) was a gift from Dr. Feng Zhang; the pCMVR8.74 (Addgene plasmid #22036) and pMD2.G (Addgene plasmid #12259) plasmids were gifts from Dr. Didier Trono [65]. SgRNA sequences (Table S6) targeting snoRNAs were based on a previous publication [66]. They were annealed and cloned into LentiCRISPRv2 using BsmBI overhangs and sequence-verified. LentiCRISPRv2 with sgRNAs targeting GFP was used as a CRISPR control [67,68]. HEK293T cells were transfected with transfer and production plasmid DNAs (12 μg DNA per 10 cm dish, second-generation lentiviral production system) in equimolar ratios using polyethylenimine (PEI, PEI/DNA ratio 2.5:1; Polysciences, 07923966-2) and lentiviruses were harvested and concentrated using the Lenti-X Concentrator (Takara, San Jose, CA, USA, 631232). Viral titers were determined by p24 ELISA (Fujirebio, Gent, Belgium, 80563) and the multiplicity of infection (MOI) was assessed by serial dilution and transduction. SW1353 cells were transduced at MOI = 1 using 8 μg/mL polybrene (Sigma-Aldrich, Zwijndrecht, The Netherlands, H9268). After 24 h, cells were selected in a medium supplemented with 2 μg/mL puromycin for 3 days (Sigma-Aldrich, P8833). SW1353 (ATCC, HTB-94) were cultured in DMEM/F-12 low glucose with GlutaMAX (Gibco Life Technologies, 31331-093) supplemented with 10% FCS (Sigma-Aldrich, F7524) and 1% A-A (Gibco Life Technologies, 15240-062). HEK293T (ATCC, CRL-3216) were cultured in DMEM high-glucose medium (Gibco Life Technologies, 41966029) supplemented with 10% FCS (Sigma-Aldrich, F7524), 1% A-A (Gibco Life Technologies, 15240-062), and 1% sodium pyruvate (Gibco Life Technologies, 11360070). Cells were cultured at 37 °C in a humidified atmosphere with 5% CO_2_.

### 4.6. DNA Isolation and Surveyor Assay 

To assess the targeting of the DNA with CRISPR/Cas9, genomic DNA (gDNA) was isolated with the DNeasy Blood & Tissue Kit (Qiagen, Manchester, UK, 69504). High-fidelity Phusion polymerase in HF buffer (Thermo Scientific, Waltham, MA, USA, F530L) and 200 ng gDNA were used for the PCR amplification of the genomic regions spanning the sgRNA target sites (primer sequences are listed in Table S6). The MinElute PCR Purification Kit (Qiagen, 28004) was used to purify and concentrate PCR amplicons. Amplified DNA (300 ng) was hybridized and digested by Surveyor Nuclease S at 42 °C (Surveyor Mutation Detection Assay IDT, 706020); fragments were separated on agarose gel (1.5%) and detected with ethidium bromide and the Chemidoc MP imaging system (Bio-Rad, Hercules, CA, USA).

### 4.7. RT-qPCR

Total RNA (600 ng) was reversely transcribed using random hexamers (Promega, Madison, WI, USA, C1181). A qPCR was performed using Takyon™ No Rox SYBR Master Mix blue dTTP (Eurogentec, Liege, Belgium, UF-NSMT-B0710), cDNA (6 ng), forward and reverse primers (300 nM), and the following protocol: 50 °C 2 min, denaturation at 95 °C 10 min, 40 cycles of amplification (15 s 95 °C and 1 min 60 °C) (Bio-Rad CFX96 Real-Time PCR Detection System). Data were analyzed using the standard curve method (Bio-Rad CFX Manager Software version 1.1). The relative quantification of target gene expression was normalized to a reference gene and gene expression data were log-transformed for the analysis. Primer sequences are listed in Table S6.

### 4.8. Proliferation Assay

Cells were seeded subconfluently (10,000 cells/cm^2^) and cultured in a proliferation medium for 6 days. On the day of sampling (Days 0, 2, 4, and 6) cells were washed with PBS, fixated with 1% glutaraldehyde (VWR, L150739.1000), and stained with 0.1% crystal violet (Sigma-Aldrich, C-3886) in 200 mM boric acid (pH 9.0) (Sigma-Aldrich, B7901). Plates were washed with deionized water, the bound dye was solubilized using 10% acetic acid (VWR, Leicestershire, UK, 20102292), and the optical density was measured at 590 nm using a Multiskan FC Microplate Photometer (Thermo Scientific).

### 4.9. Total Protein Synthesis

*SNORA33* KO and CRISPR control cell pools were cultured for 30 min in methionine and cysteine-free medium supplemented with EasyTag™ EXPRESS 35S Protein Labeling Mix (25 μCi/mL; PerkinElmer Waltham, MA, USA, NEG772002MC) and harvested by scraping in RIPA buffer. A Tri-Carb 2910 TR scintillation counter (PerkinElmer) was used to measure the radioactive signal and data were normalized to the total protein content of the well, as determined by a standard BCA assay (Sigma-Aldrich, 71285-3).

### 4.10. Polysome Profiling

*SNORA33* KO and CRISPR control cell pools at 70–80% confluency were pre-incubated with cycloheximide (CHX, 100 µg/mL, Sigma-Aldrich, C1988) for 20 min at 37 °C, harvested by scraping in cold NaCl (0.9%) supplemented with CHX (100 µg/mL), centrifuged at 1.100 rpm (Hettich Rotanta 460, Tuttlingen, Germany) for 10 min, and gently resuspended in polysome extraction buffer (20 mM Tris–HCl (pH 7.5), 100 mM KCl, 5 mM MgCl_2_, 0.5% NP-40, CHX 100 μg/mL, protease inhibitor cocktail (Sigma-Aldrich, 11836170001), RNasin (40 U/mL, Promega, N2515)) [69] and incubated on ice for 10 min. To remove nuclei and cellular debris, cytoplasmic extracts were centrifuged at 13.200 rpm (Hettich Micro 200R, Tuttlingen, Germany) at 4 °C for 10 min. Linear sucrose gradients (10–50%) were prepared in open-top Polyclear tubes (Senton, 7030) by layering and mixing an equal amount of 50% and 10% sucrose solutions (0.5 M NaCl, 100 mM Tris–HCl (pH7.5), 50 mM MgCl_2_) using a Gradient Master 108 (BioComp). Equal amounts of cytoplasmic extracts were loaded onto the sucrose gradients and centrifuged at 39.000 rpm at 4 °C for 1.5 h (SW41 Ti Swinging-Bucket Rotor, Beckman-Coulter Inc, Indianapolis, IN, USA, 331362) [69]. A piston gradient fractionator (BioComp) coupled to a fraction collector (Gilson) and continuous A260 monitoring (Triax Flow Cell, Science Services, Munich, Germany) were used to separate sucrose gradient fractions (24 fractions, 500 μL each). 

### 4.11. Translational Modus and Fidelity Assays

To assess ribosome translation characteristics, IRES-mediated translation initiation and translation fidelity (stop codon skipping and frameshift) were measured using dual-luciferase reporters (DLRs; gifts from Dr. S.R. Thompson and Dr. J. Dinman, respectively; sequences in Table S6). Plasmid DNA was transfected with Fugene6 Transfection Reagent (Promega, E2691; Fugene:DNA 4:1, 0.5 μg DNA/well of 24-well plate). Cells were co-transfected with lacZ plasmid (10% of transfected DNA). After 4 h of incubation, the medium was supplemented with 2% A-A culture medium and incubated for an additional 20 h. Firefly and Renilla luciferase activity were measured 48 h post-transfection by the Dual-Luciferase^®^ Reporter (DLR™) Assay System (Promega, E1910) and a Tristar2 LB942 (Berthold Technologies). The β-galactosidase activity was measured with the β-Gal Assay Kit (Invitrogen, K1455-01) and a Multiskan FC Microplate Photometer (Thermo Scientific). Raw RLU values are listed in Table S7 [70]. The Fluc/Rluc ratio was corrected for the β-galactosidase activity of the well.

### 4.12. Label-Free Liquid Chromatography–Tandem Mass Spectrometry (LC-MS/MS) Analysis of Cellular and Ribosomal Proteome

For the analysis of the cellular proteomes, *SNORA33* KO and CRISPR control cell pools were harvested at 70–80% confluency by scraping in a 25 mM ammonium bicarbonate buffer (Sigma-Aldrich, 09830) supplemented with 7.5U of Benzonase Nuclease (Merck Millipore, Darmstadt, Germany, 70664-3) and a protease inhibitor (Sigma-Aldrich, 11836170001). Samples were sonicated on ice and centrifuged, and supernatants were flash-frozen in liquid nitrogen. For the analyses of the ribosomal proteome, *SNORA33* KO and CRISPR control cell pools were pre-incubated with CHX (100 µg/mL, Sigma-Aldrich, C1988) for 5 min at 37 °C and harvested by scraping in cold NaCl (0.9%) supplemented with CHX (100 µg/mL). Samples were spun at 1.100 rpm (Hettich Rotanta 460) for 5 min and gently resuspended in cytoplasmic extraction buffer (20 mM Tris pH 7.5, 5 mM MgCl_2_, 10 mM NaCl, 0.15% NP-40), freshly supplemented with CHX (100 µg/mL), a protease inhibitor cocktail (Sigma-Aldrich, 11836170001), and recombinant RNasin (Promega, N2515). Cells were lysed using a Dounce homogenizer (50 strokes/sample). To remove nuclei and mitochondria, cytoplasmic extracts were centrifuged first at 3.900 rpm (Hettich Micro 200R) at 4 °C for 10 min and afterwards at 14.000 rpm (Hettich Micro 200R) at 4 °C for 10 min. Equal amounts of cytoplasmic extracts were loaded on low-salt sucrose cushions for the isolation of ribosomes and associated proteins (1 M and 0.7 M sucrose cushion; 20 mM Tris pH 7.5, 5 mM MgCl_2_, 25 mM NaCl) [15] and ultracentrifuged at 32.200 rpm at 4 °C for 17 h (SW 41 Ti rotor, Beckman-Coulter, 331362). The ribosomal pellet was resuspended in 25 mM ammonium bicarbonate buffer (Sigma-Aldrich, 09830) supplemented with 7.5U of Benzonase Nuclease (Merck Millipore, 70664-3) and a protease inhibitor (Sigma-Aldrich, 11836170001) and flash-frozen in liquid nitrogen. In-solution tryptic digestion of the cell lysates was performed as previously described [71]. In short, 25 mM ammonium bicarbonate (Fluka Chemicals Ltd., Gillingham, UK) containing 0.05% (*w*/*v*) RapiGest (Waters, Elstree, UK) was added to 100 μg protein of each sample to produce a final volume of 160 µL and heated at 80 °C for 10 min. DL dithiothreitol (Sigma-Aldrich) was added (3 mM final concentration) and incubated at 60 °C for 10 min. Iodoacetamide (Sigma-Aldrich) was added (9 mM final concentration) and incubated at room temperature for 30 min in the dark. Tryptic digestion was undertaken using Lys-C endopeptidase (FUJIFILM Wako Pure Chemical, Osaka, Japan) at a ratio of 1:25 177 (enzyme:protein) and incubated at 37 °C for 16 h, followed by a second trypsin supplementation for 2 h. Digestion was halted by the addition of trifluoroacetic acid (Sigma-Aldrich) to 0.5% (*v*/*v* final concentration) and rotation for 30 min at 37 °C. Samples were centrifuged at 4 °C at 13,000 rpm for 10 min and the supernatant was removed. Five hundred ng of each tryptic digest was analyzed using LC-MS/MS, on a 2 h gradient for the cellular proteome and a 30 min gradient for the ribosomal proteome. Data analyses were performed as previously described [71]. The Progenesis QI software (V4, Waters) was used for protein quantification (only unique peptides were considered) [72]. Data are available via ProteomeXchange with the identifier PXD042545. We used Reactome to categorize all identified proteins in the ribosomal proteome [41].

### 4.13. Statistical Testing

All statistical tests were performed using GraphPad Prism 5.0.1. (San Diego, CA, USA). A normal distribution was tested or assumed (indicated in figure legends). Differences between the two groups were determined by a two-tailed unpaired *t*-test or Mann–Whitney U test. Two paired groups were analyzed by a two-tailed paired *t*-test. The LC-MS/MS cellular and ribosomal proteome data were log-transformed and analyzed by one-way analysis of variance (ANOVA). Additional details regarding the statistical analyses are indicated in the figure legends. Data are presented as mean ± SD. Ns—not significant, * *p* < 0.05, ** *p* < 0.01, *** *p* < 0.001.

## Data Availability

Proteomic data are available via ProteomeXchange with the identifier PXD042545.

## Supplementary Materials

The following supporting information can be downloaded at: https://www.mdpi.com/article/10.3390/ijms241612578/s1.

## Abbreviations

2′-O-me	2′-*O*-methylation
A-A	antibiotic–antimycotic
ANOVA	analysis of variance
ANS	anisomycin
AP1G1	adaptor related protein complex 1 subunit gamma 1
BCA	bicinchoninic acid
CHX	cycloheximide
CPM	counts per minute
DE	differentially expressed
DLR	dual-luciferase
DMEM/F-12	Dulbecco’s modified eagle medium/nutrient mixture F-12
dTTP	deoxythymidine triphosphate
ELISA	enzyme-linked immunoassay
EMT	emetine
FA	fucidic acid
FBL	fibrillarin
FC	fold change
FCS	fetal calf serum
gDNA	genomic DNA
GFP	green fluorescent protein
sgRNA	single guide RNA
HAC	human articular chondrocyte
KCl	potassium chloride
KO	knockout
M	male
MgCl_2_	magnesium chloride
NaCl	sodium chloride
NPM1	nucleophosmin
nm	nanometer
OA	osteoarthritis
OA-SF	osteoarthritic synovial fluid
PBS	phosphate-buffered saline
RLU	relative light unit
RIN	RNA integrity number
RIPA	radioimmunoprecipitation assay
rRNA	ribosomal RNA
RT-qPCR	quantitative reverse transcription polymerase chain reaction
snoRNA	small nucleolar RNA
