# Supplementary material for: Depletion of SNORA33 Abolishes ψ of 28S-U4966 and Affects the Ribosome Translational Apparatus
Source: Int J Mol Sci. 2023 Aug 8;24(16):12578. doi: 10.3390/ijms241612578 (PMC10454564; doi:10.3390/ijms241612578)
Supplement: Supplementary file 1 [file ijms-24-12578-s001.zip › Supplementary figures_proof.pdf]

Supplementary figures

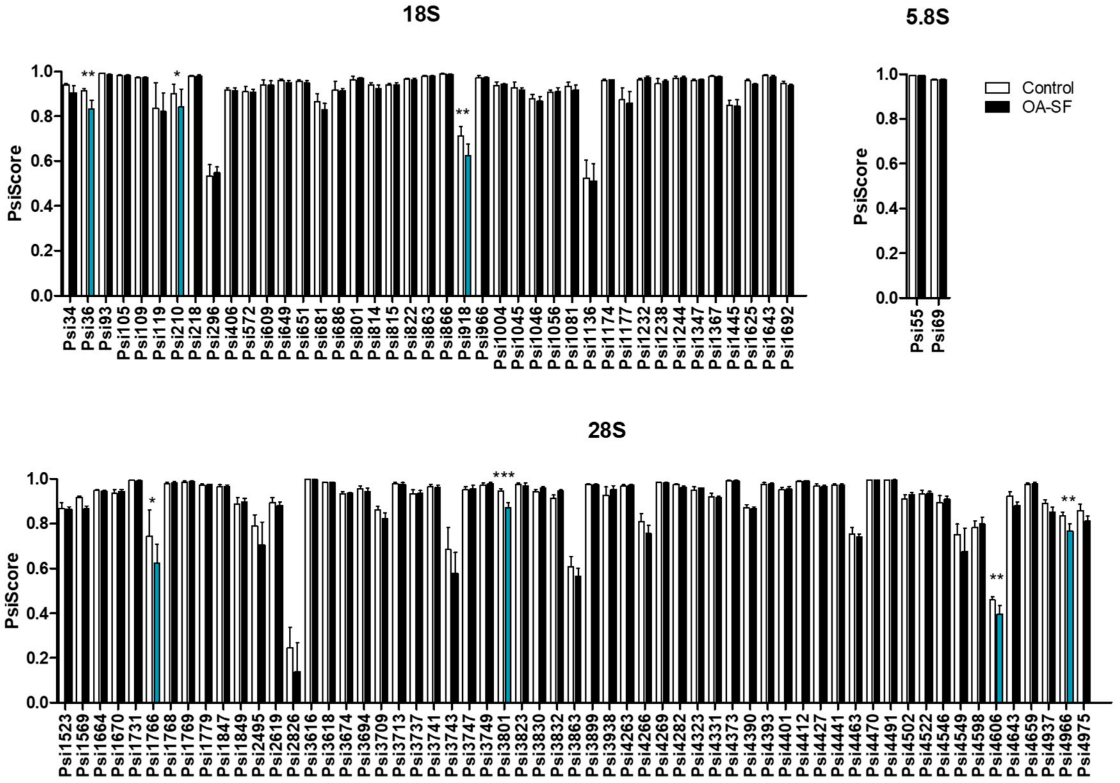

Figure S1: Ribosomal RNA pseudouridylation profile of human articular chondrocytes exposed to a chronic disease microenvironment. Non-OA HACs of 5 individual donors were exposed to a chronic disease microenvironment represented by the synovial fluid from end-stage knee osteoarthritis patients (OA-SF, pool of 14 donors, 20% (v/v)) for 14 days. Levels of pseudouridylation of human rRNAs were evaluated by HydraPsiSeq. Differentially ψ sites are indicated in green. Table S3 lists the PsiScore values of all rRNA ψ sites in control and OA-SF treated samples. 28S numbering (GenBank sequence U13369.1). * $p < 0.05$, ** $p < 0.01$, *** $p < 0.001$.

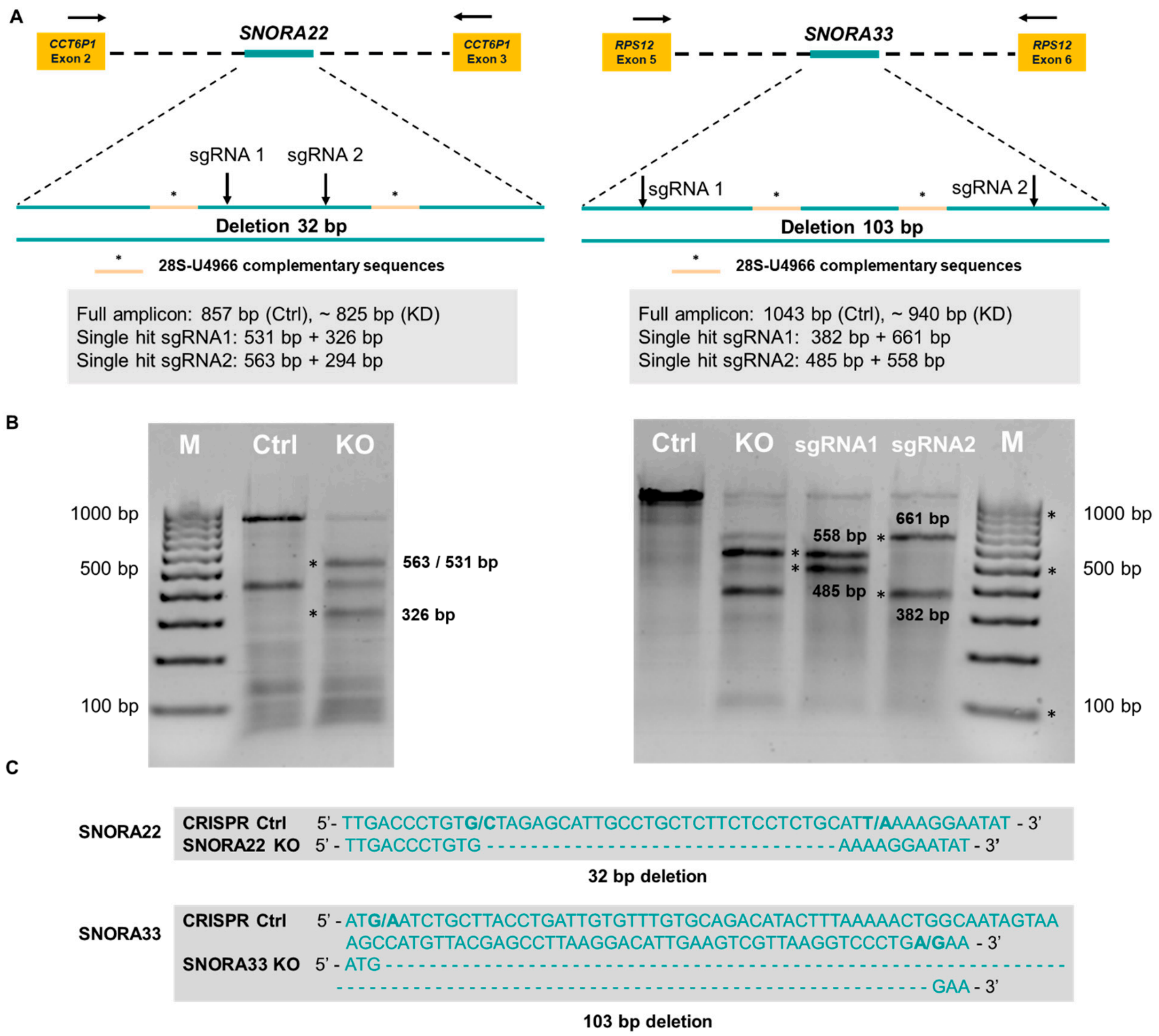

Figure S2: Double sgRNA targeting approach to generate *SNORA22* and *SNORA33* SW1353 KO cell pools. (A) Schematic of the double sgRNA targeting approach to generate the *SNORA22* and *SNORA33* KO SW1353 cell pools. Top: Exons of the host genes *CCT6P1* and *RPS12* flanking the intron-encoded *SNORA22* and *SNORA33* respectively. Arrows represent genomic PCR primers used to amplify the targeted regions. Middle: Scheme of *SNORA22* and *SNORA33* sequences with indicated sgRNA1 and sgRNA2 cleavage sites and 28S-U4966 complementary sequences. Double lentiviral transduction with sgRNA1 and sgRNA2 was performed in SW1353 cells separately for *SNORA22* and *SNORA33*. Bottom: Overview of amplicon sizes and expected Surveyor Nuclease S digestion products. (B) Surveyor assay results. Marker DNA (M; 100 bp DNA Ladder), CRISPR control (Ctrl), *SNORA22* / *SNORA33* KO cell pools. Bands of interest are indicated with an asterisk (*). (C) Schematic of the genomic modification in the cell population assessed by sequencing. SgRNA1 and sgRNA2 cleavage sites are in bold and indicated by a slash.

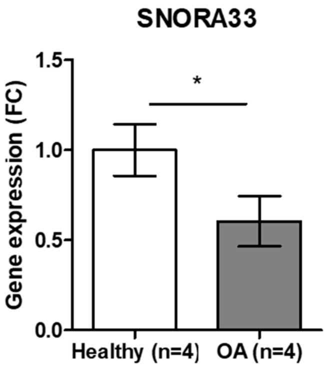

Figure S3: Relative expression of *SNORA33* in human primary chondrocytes. The expression levels were measured by RT-qPCR (n=4, individual donors) and normalized to the reference gene (*PPIA*) expression. Data were analyzed by unpaired t-test with the assumption of normal distribution. * p < 0.05.

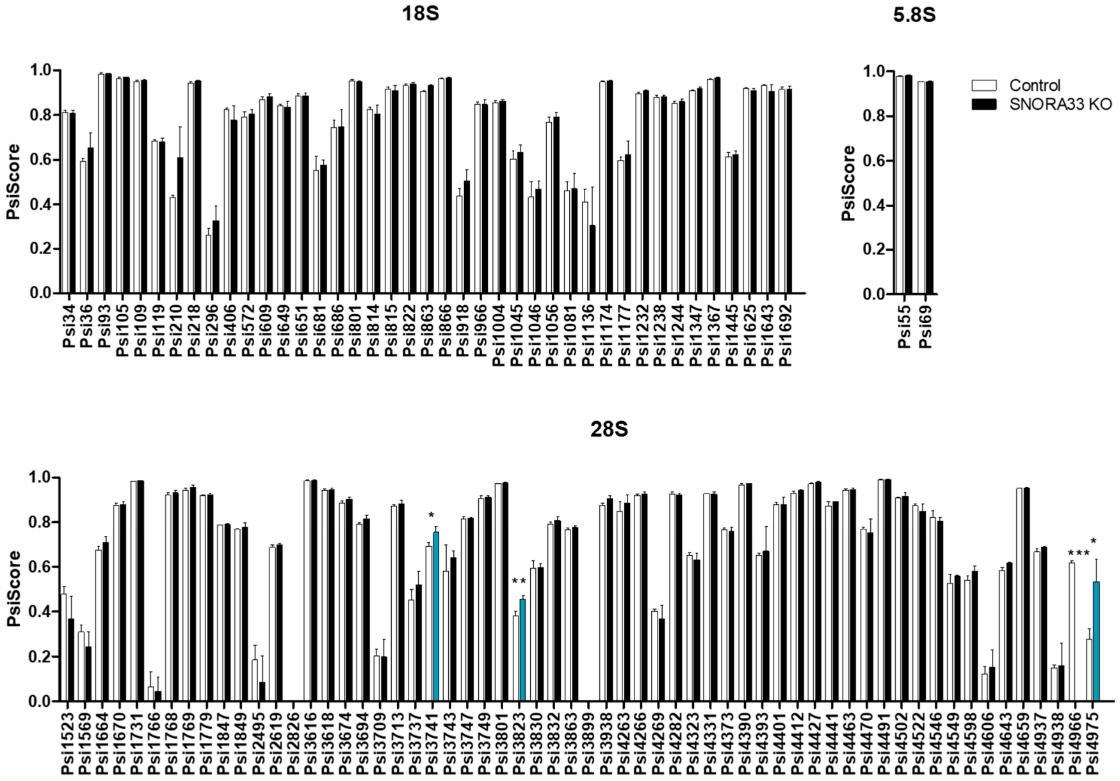

Figure S4: Ribosomal RNA pseudouridylation profile of *SNORA33* KO cell pool. Levels of pseudouridylation of human rRNAs were evaluated by HydraPsiSeq (n=3). Differentially ψ sites are indicated in green. Table S4 lists the PsiScore values of all rRNA ψ sites in *SNORA33* KO and CRISPR Ctrl cell pools. * p < 0.05, ** p < 0.01, *** p < 0.001.

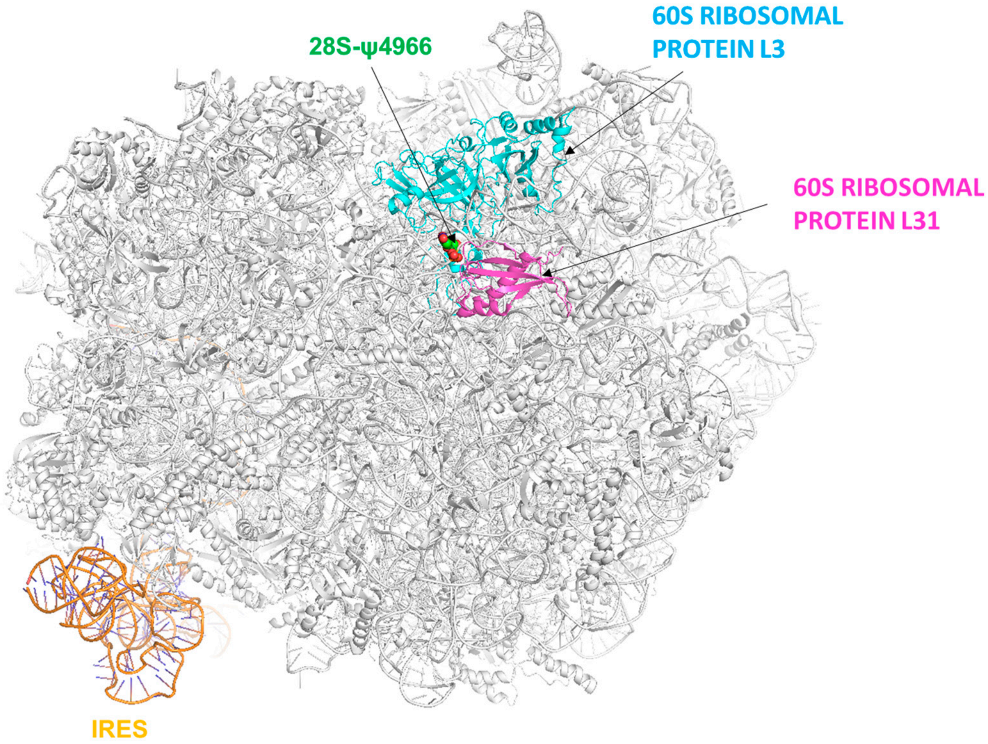

Figure S5: Binding of HCV IRES to the human ribosome relative to the position of 28S rRNA nucleotide 4966 (PDB ID: 4UJD, mammalian 80S HCV-IRES initiation complex with eIF5B PRE-like state).
